# Medical faculty profile is an important determinant of student profile and future practice expectations of medical students in Angola

**DOI:** 10.1186/s12909-021-02836-z

**Published:** 2021-09-01

**Authors:** Inês Fronteira, Helga Freitas, Nkanga Guimarães, Mário Fresta, Paulo Ferrinho

**Affiliations:** 1 Global Health and Tropical Medicine, Instituto de Higiene e Medicina Tropical, Universidade Nova de Lisboa, Lisbon, Portugal; 2 Ministério de Saúde, Luanda, Angola; 3 Luanda, Angola; 4 CEDUMED, Universidade Agostinho Neto, Luanda, Angola

**Keywords:** Angola, Medical students, Professional expectations, Profile

## Abstract

**Background:**

Angola is among one of the most deprived countries in the world in terms of medical professionals. In the past decade, the Angolan Government has invested in the expansion of faculties of medicine in the country.  We analysed the profiles of medical students in Angola according to four clusters of medical schools: older faculty in the country, private faculties, Cuban sponsored faculties and military faculty; under the assumption that the organizational culture of the different faculties might influence the expectations and decisions towards future professional life of medical students regarding where they want to work (community versus hospital) and in which sector (exclusively public versus not exclusively public).

**Methods:**

Observational cross-sectional study. Piloted, standardized questionnaire to final year medical students or higher year of training in the first four-month of 2014 (*N* = 402). Data were entered into a SPSS v.20 database and descriptive statistics computed. Statistical significance for categorical variables was tested by Pearson chi-square, Fisher exact or likelihood ratio tests as appropriate. Comparison of means was tested with Anova. Backward elimination binary logistic regression was used to test the hypothesis that type of faculty of medicine is an important determinant of future professional practice, i.e., level (hospital vs. community) or sector of practice (exclusive public sector vs. private or private and public), while controlling for confounders.

**Results:**

After controlling for age, sex, marital status, place of birth and place of primary and secondary education, type of family and family influence, students were more likely to choose community over hospital practice and to prefer exclusive public practice if attending a Cuba supported faculty of medicine.

**Conclusions:**

Medical education cannot be isolated from planning of the medical workforce. Some important and impactful careers choices, like choosing rural over urban practice, public over private sector practice, have deep influences in the medical professionals’ labour market. Some of these decisions are shaped even before the end of the medical training. As such, the monitoring of future professional intentions in medical schools should be done regularly to accommodate both the health system needs and the hopes and dreams of medical trainees.

## Key messages

- Monitoring of the medical students preferences is important for medical workforce planning.
- Training in underserved areas seems to favour intention to work in those areas.
- Rurality of medical students is important for future community practice.
- Training in Cuban medical faculties is associated with greater chance of working in the public sector.

## Background

Angola, a country on the Atlantic coast of sub-Saharan Africa, has about 33 million inhabitants. Currently, there are just over 6,000 doctors, more than 900 are foreigners and about 3,000 are in the public sector, making it one of the countries with a severe shortage of medical professionals (18.2 medical doctors per 100 000 inhabitants) [1].

The strengthening of the medical workforce, and of the health workforce in general, cannot be achieved without a strong and strategic connection with the educational sector which shares budget restrictions and understaffing with the health sector. In 2010 the Angolan government spent 3.4 % of the GDP with education and in 2006 – last year available – 9 % of that value was spent with tertiary education [2].

Nevertheless, there has been an effort, throughout health workforce severely deprived countries, to address medical workforce shortages, to which Angola is not an exception [3].

In this country, medical education has tried to keep up with the changes and needs in the health care system. Angola has a medical faculty since colonial times (known today as Faculdade de Medicina da Universidade Agostinho Neto, FM-UAN). Following independence (in 1975), this faculty continued producing doctors to partially meet the needs of an exclusively public sector, socialist health care system. Subsequent efforts were made to adapt medical curricula to a new vision of a society where, since 1992, other social partners emerged as providers of health care and as trainers of medical students. At the turn of the millennium, two private faculties of medicine (FM) opened in Luanda (FM-P). With Cuban support, a number of public sector FM (FM-C) have been opening in provincial capitals: 3 in 2008, 2 in 2009, and 2 others in 2016. With the support of Portuguese and Cuban FM, the Ministry of Defense opened its own medical faculty in 2008 (FM-D) [4–6].

Currently there are 11 FM, 4 [FM-UAN (*n* = 1), FM-P (*n* = 2) e FM-D (*n* = 1)] located in the capital city, Luanda, with the remaining (FM-C) distributed per 7 provinces (2 of these were created after the study described in this article).

All courses confer the same degree of “licenciado em medicina” (basic medical education) and last six years, with candidates being selected through a competitive exam, designed and applied in each one of the faculties. The FM-C offer the same program and end with a final examination, while the others have the public defense of a final course monograph. All courses end with a (professional) internship that lasts an entire year, except for one of the private faculties where it is restricted to 200 h. In public faculties there is no tuition. In private faculties, annual tuitions varied between Kz 385,000 and 480,000 (approx. 595 USD and 742 USD).

As distinctive features, apart from “traditional disciplines”, the FM-UAN develops competencies in public health throughout the course (introduction to public health, statistics, hygiene, epidemiology, demography, community health, health administration); FM-D offers history of medicine, biostatistics and informatics, research methodology and, for two years, English; the FM-C offer physical education for two years and English for five years. One of the private courses offers philosophy, Portuguese, Latin, social sciences, computer science, anesthesiology, clinical experimentation, ethics & deontology; and the other biostatistics & computer science, research methodology, English, Portuguese, bioethics, social sciences, first aid and health management.The current ratio of FM per population is 1 per 700,000 population, higher than the recommended ratio of 1 per 2 million population [7].

The medical school environment reflects the values of the educational culture, its beliefs and behaviors that comprise a powerful array for the construction of the professional identity and of career choices for students and to which doctors constantly return to during their working life [8–16].

It is believed that the organizational culture of the different Angolan FM currently training future medical doctors are different and that these differences result, in part, from differences among the students, namely in terms of baseline characteristics and also from the sponsoring entity (University Agostinho Neto, the Army, the private sector, or the Cuban cooperation).

This study compares the profiles of highest year of training of medical students in the four different groups of FM, according to the sponsoring entity. The study used data collected for the preparation of the Health Work Force Strategic Plan 2014–2025 for the Angolan Government, developed in 2013–2014.

## Methods

Observational cross-sectional study. The population of the study comprised all FM (private and public) of Angola in existence in 2014. All 9 adhered to the study except 1 FM-C. No sample was drawn.

A piloted, standardized questionnaire, with closed and open-ended questions, was distributed to all final year medical students or higher year of training in the studied faculties (Table 1), during an agreed lecture period in the first four-month of 2014. The majority of students were in their 6th year of training (out of a total of seven).

**Table 1:** Distribution of students per medical faculty and year of training

Year of training	FM-UAN	FM-C	FM-D	FM-P	Total
5th	0	26 14 %	0	4 5 %	30 8 %
6th	108 98 %	154 86 %	30 100,0 %	66 86 %	358 90 %
7th	2 2 %	0	0	7 9 %	9 2 %

The administration of the questionnaire was subcontracted to a commercial firm. The questionnaire applied was similar to the one applied in other studies of medical students in Portuguese speaking countries [5, 17–21].

Data were entered into a SPSS v.20 database and descriptive statistics (counts, frequencies, mean and standard deviation and medians) were computed. Statistical significance for cross-tabulation of categorical variables was tested by Pearson chi-square, Fisher exact or likelihood ratio tests as appropriate. Comparison of statistical significance of means was tested with Anova [22]. Backward elimination binary logistic regression [23] was used to test the hypothesis that type of FM (UAN, P, C or D) is an important determinant of future professional practice expectations, namely in terms of level of practice (hospital vs. community) or sector of practice (exclusive public sector vs. private or private and public sector), while controlling for potentially confounding variables. Variables included in the logistic regression comprised those suggested in literature and/ or those found to have a statistical association with a p-value < 0.1.

## Results

A total of 402 students answered the questionnaire. Student distribution was: 45 % (*n* = 180) from FM-C, 28 % (*n* = 114) from FM-UAN, 19 % (*n* = 78) FM-P and 8 % (*n* = 30) from FM-D.

### Socio-demographic characteristics of students

The mean age of medical students in the study was 29.0 ± 5.4 years, the oldest group belonging to FM-UAN students (31.1 ± 7.1 years of age) and the youngest to FM-D (25.6 ± 1.5); FM-C and FM-P had very similar averages (28.6 ± 4.1 and 28.4 ± 5.1 respectively) (*p* < 0.01).

The feminization of the student corps was highest for the FM-C (91 %), followed by FM-P (72 %), FM-UAN (62 %) and least for FM-D (53 %) (*p* < 0.01).

Most students were single and, except for FM-UAN, from families with rural background. Students from FM-UAN were most frequently married and predominantly from urban families (Table 2).

**Table 2:** Distribution of socio-demographic characteristics of students per medical faculty

Variables		FM-UAN	FM-C	FM-D	FM-P	*p*-value
Age	Mean (sd)	31.1 (7.1)	28.6 (4.1)	25.6 (1.5)	28.4 (5.1)	< 0.01
Gender	Female	70 62 %	108 91 %	16 53 %	56 72 %	< 0.01
	Male	43 48 %	11 12 %	14 16 %	22 24 %	
Marital status	Single	62 56 %	18 64 %	26 87 %	63 81 %	0.012
	Married	47 42 %	10 36 %	4 13 %	15 19 %	
	Separate/ Divorced	1 1 %	0 0 %	0 0 %	0 0 %	
	Widowed	1 1 %	0 0 %	0 0 %	0 0 %	
Family	Urban	63 58 %	70 40 %	12 42 %	30 40 %	0.04
	Rural	4 4 %	8 4 %	3 10 %	6 8 %	
	Mixed urban/ rural	41 38 %	98 56 %	14 48 %	40 52 %	
Primary school education	Luanda	7 63 %	20 11 %	14 47 %	50 66 %	< 0.01
	Other provincial capital	31 27 %	127 72 %	16 53 %	15 20 %	
	Other place in Angola	9 8 %	30 17 %	0 0 %	4 5 %	
	Abroad	2 2 %	0 0 %	0 0 %	7 9 %	
Secondary school education	Luanda	78 68 %	12 7 %	14 47 %	53 70 %	< 0.01
	Other provincial capital	27 24 %	144 82 %	14 47 %	12 16 %	
	Other place in Angola	6 5 %	20 11 %	0 0 %	3 4 %	
	Abroad	3 3 %	0 0 %	0 0 %	8 11 %	

Most students from FM-UAN and FM-P, both based in Luanda, were born in the capital city (52 and 58 % respectively). Although based in Luanda, FM-D had a larger diversity in terms of students, with the majority coming from outside Luanda (only 30 %, were from Luanda), as did the FM-C (only 8 % from Luanda) (*p* < 0.01) (Table 2).

The same pattern prevailed for the association between the type of FM and place of completion of primary or secondary education (Table 2). However, for place of completion of secondary education, the exception previously found for FM-D was not noted anymore – for all faculties, completing secondary education in the city of location of the faculty seems an important entrance association (Table 2).

70 % (*n* = 14/20) of students from rural families were born in a capital of one of the provinces (other than Luanda) compared with only 54 % (94/173) and 55 % (106/192) for urban or mixed urban and rural families (*p* < 0.05).

### Decision about studying medicine

More than half of the students (62 %) had relatives that were health professionals. This was particularly evident for those attending FM-C (Table 3).

**Table 3:** Distribution of variables related with the decision to study medicine per type of faculty of medicine

		FM-UAN	FM-C	FM-D	FM-P	*p*-value
Family members in health professions (yes)		66 59 %	125 69 %	12 40 %	45 58 %	0.01
This course was your first choice (yes)		105 94 %	168 93 %	25 83 %	71 91 %	0.25
Would choose same course if could go back (yes)		87 78 %	177 98 %	16 53 %	64 83 %	< 0.01
Family’s influence on the choice of studying medicine	none	36 33 %	48 27 %	7 25 %	23 32 %	0.44
	some	29 26 %	57 32 %	12 43 %	16 22 %	
	strong	46 41 %	72 41 %	9 32 %	33 46 %	

Relatives were an important influence in the choice of the medical degree for 71 % of the students, more so for the students attending the military faculty (FM-D) and the FM-C.

The choice for medical training was the first choice for 92 % of the students, but except for the FM-D, less than a quarter would choose a different course if they could go back (Table 2). The decision to study medicine was taken at average age of 15.5 ± 5.8 years with no significant differences between faculties.

### Student performance

Less than 1 % of the students were repeating the current year of training (*n* = 4) or had accumulated curricular units from previous years (*n* = 2) (differences between faculties were not significant).

### Expectations regarding future medical practice

Except for FM-C, where students favor exclusive public sector practice and have the highest tendency to practice at community level, students from the other clusters of faculties prefer hospital and dual public and private practice, more in those from FM-D. Only 3 students from FM-P wanted to practice exclusively in the private sector (data not shown in table). Again, most students wanted to practice in the capital of the province where they were doing their medical studies (Table 4).

**Table 4:** Future practice preferences of students

		FM-UAN	FM-C	FM-D	FM-P	*p*-value
Sector of preferred practice after completing training	public	51 45 %	112 64 %	7 23 %	34 45 %	< 0.01
	both or just private	61 55 %	64 36 %	23 77 %	41 55 %	
Level of preferred practice after completing training	community	15 14 %	55 33 %	4 14 %	10 16 %	0.01
	hospital	91 86 %	113 67 %	25 86 %	54 84 %	
Place of preferred practice after completing training	Luanda	83 76 %	14 8 %	19 64 %	54 84 %	< 0.01
	Other provincial capital	24 22 %	142 82 %	10 33 %	15 21 %	
	Other locality	2 2 %	16 9 %	1 3 %	0 0 %	
	Abroad	1 1 %	1 1 %	0 0 %	3 4 %	

After controlling for age, sex, marital status, place of birth and place of primary and secondary education, type of family and family influence, students were more likely to choose community over hospital practice (Table 5) and also more likely to prefer exclusive public practice (Table 6) if attending FM-C. Being from families with rural connections or being trained in one of the Cuban supported faculties increased the odds of preference for future community practice over hospital practice. Increasing age and, again, being trained in one of the Cuban supported faculties favored a greater interest for public practice (Table 6).

**Table 5:** Determinants of preference for future community practice

Variables	aOR	95 % C.I. for aOR
Predominantly rural families (reference predominately urban)	1.337	[1.020;1.754]
Type of faculty (reference FM-P)		
FM-UAN	0.970	[0.403;2.336]
FM-C	2.494	[1.171; 18.485]
FM-D	0.635	[0.160; 2.532]

**Table 6:** Determinants of preference for future public practice vs. not exclusively public practice

	aOR	95 % C.I. for aOR
Type of faculty (compared with FM-P)		
FM-UAN	0.795	[0.427;1.481]
FM-C	2.000	[1.139;3.509]
FM-D	0.430	[0.162; 1.143]
Age (years)	1.051	[1.010; 1.100]

## Discussion

Human resources for health are paramount to deliver safe, timely and quality health care. The Global Strategy on Human Resources for Health recognizes that HRH are cornerstone to achieve 5 universal health coverage and, consequently, to attain the Sustainable Development Goals [24].

Not overlooking the importance of other health professionals, Governments are struggling to scale-up the number of medical professionals, especially in underserved and complex scenarios. Training medical professionals or, for that matter, health professionals in general, is not only a problem of funding but a fragile equilibrium between being able to maintain the provision of medical services, while, at the same time, increasing the training capacity using already scarce and overworked resources [25].

After training medical professionals, the challenges are even greater. Deployment of medical professionals to where they are needed or assuring a good distribution between served and underserved areas, hospital and community level and managing competition between public, private and social sectors for a scarce resource are some of these challenges, along with brain drain, turnover and migration [26–28].

Angola is among one of the most deprived countries in the world in terms of medical professionals. Nevertheless, in the past decade, the Angolan Government has made an evident effort to scale-up the number of newly graduated medical professionals by investing in the expansion of FM in the country [6]. Nowadays there are 11 FM in Angola: FM-UAN, the oldest one in the country, two private and one associated with the Ministry of Defence, all in Luanda, and the remaining seven, supported by Cuban FM, spread through the capitals of the provinces of Angola.

In this study, we analysed the profiles of medical students in Angola according to four clusters of medical schools: FM-UAN, FM-P, FM-C and FM-D under the assumption that the organizational culture of the different FM, that partially result from the students’ characteristics and from the sponsoring entities, might influence both their expectations and decisions towards future professional life. In this last case, we examined two future decisions that medical students must take once they finish their training – where they want to work (community versus hospital) and in which sector they want to work (exclusively public versus not exclusively public).

We collected data from students attending the Angolan medical schools, preferably those in their last year of training, in 2014. However, in some of the FM, because they had started operating recently, students were not in their last year of training. Because professional expectations and future intentions are contextual and somehow volatile, we believe that students might change their minds later during their training. As so, results must be interpreted bearing this in mind.

The year when the study was conducted (2014), in relation to the present time, might raise some questions in terms of the changes that meanwhile have occurred. However, since 2014, when five new public medical training courses were created in cooperation with Cuba, the situation has little evolved given the profound and long-lasting crisis that hit Angola. The number of courses remains the same, no new personnel was admitted, nor any significant changes occurred, reason why 2014 remains the most significant and recent benchmarking of medical education in the country.

We were not able to check the response rate per faculty or to which percentage of medical students did the sample in this study corresponded to, since we accessed data collected for developing the HRH strategic plan in Angola during 2014, and we do not have information on all the details of the data collection, contracted out to a firm specialized in surveys. Despite acknowledging this as a limitation of the study, we believe that the conclusions drawn from this study are valid and should be considered when deciding on the strategy to scale up the training of medical professionals and to retain them where they are most needed.

After controlling for differences between the students of the four clusters of FM, belonging to a predominantly rural family and attending a Cuban supported medical faculty increased the odds of choosing to work in the community over of the hospital.

The FM-C are based in the capital of the provinces of Angola, in which rurality is more striking. On the other hand, the curricula of these FM tend to have a bigger focus on community-based education and service, which has been described as one of the determinants for preferring rural over urban practice [8–10, 16]. The results of this study are also in line with the current evidence that describes students that come from a rural family/background as being more prone to choose a career in a rural area where a more general and comprehensive medicine practice occurs, than those from predominantly urban families [29]. Actually, having a rural background and contact with rural contexts during medical training have been described as part of the rural pipeline in medical practice (i.e., contact between rural secondary schools and the medical profession; selection of rural students into medical programs; rural exposure during medical training; and measures to address retention of the rural medical workforce), a set of strategies that can increase retention of medical professionals in rural settings [30]. In a country where the majority of medical professionals are concentrated in urban areas, this finding can help tailor medical training policies to address the needs of the health system and of the population.

Students from FM-C and who were older preferred the public sector for future practice over the private sector.

One of the possible explanations is that as FM-C are located outside the capital city Luanda, where the private sector has more expression. Students who attend these faculties do not contact so closely with the private sector and, as so, do not consider working there after graduation. The structure of the market for physician services as well as supply and demand factors have been described as influencing the decision to engage in dual practice or not exclusively public practice [31]. On the other hand, living in Luanda might demand a higher income and students can consider the private sector as a way to increase income, which is the case for all medical students not affiliated with the FM-C [32]. The average annual income for a doctor in the public sector is approximately 36,784 USD. The majority of doctors works in the public sector and engages in dual practice. This overlap of public and private practices, especially those in urban areas, leads to great variations in income (e.g., from 1250 USD per months for a non-specialized medical doctor working 2 to 3 times per week to several thousands of dollars for surgeons).

The findings of this study seem to contribute to the argument that the geographical location of medical schools should be thoroughly considered in HRH strategy, especially in terms of future deployment and retention of medical professionals. The characteristics of the health market (e.g., weaker or stronger private sector, demand and supply of health services), rurality and students’ background, as well as the proximity to the community should be analysed when planning to scale-up medical education and medical professionals.

A final observation regarding family influences in the choice of medical studies. Family influences weighed most in Cuban supported faculties and in the military institution. The reason for this is speculative but in more rural contexts, as we expect the provincial capitals where FM-C are based to be, family influences are stronger than in metropolitan contexts [33]. For the military set up we might have two sets of influences: family members in the health professions and family members in the military. It has been described that parental and family variables are strong influences regarding the choice to join and remain in the military [34]. This is a reflection of social reproduction mechanisms (i.e. perpetuation of social systems) at work [35, 36]. Similar social reproduction mechanisms might explain the least marked feminization of the student corps of the military, as women who join the armed forces are faced with an environment designed by and for men and still face many forms of discrimination [37] .

## Conclusions

Medical education cannot be isolated from planning of the medical workforce. Some important and impactful careers choices that, at a first glance, seem to be individual choices, like choosing rural over urban practice, public over private sector practice, have deep influences in the medical professionals’ labour market. Additionally, some of these decisions are shaped even before the end of the medical training.

## Recommendations

The monitoring of future professional intentions in medical schools should be done regularly to accommodate both the health labor market needs and the expectations, hopes and dreams of medical trainees.

## Data Availability

The dataset generated and/or analysed during the current study are not publicly available because they were generated during an official support mission to the Government of Angola but are available from the corresponding author on reasonable request.

## Abbreviations

FM: Faculties of medicine
FM-UAN: Faculty of Medicine, Universidade Agostinho Neto
FM-P: Private medical faculties
FM-C: Cuban supported medical faculties
FM-D: Faculty of medicine of the Ministry of Defense
HRH: Human resources for health
